# Psychological Burden and Coping Strategies Among Pakistani Adults: A Cross-Sectional Survey Study

**DOI:** 10.3390/epidemiologia6030030

**Published:** 2025-06-20

**Authors:** Madeeha Malik, Humaira Rehman, Azhar Hussain, Ayisha Hashmi, Khalid Ahmad Al-Sunaidar, Georgina Balogh, Márió Gajdács, Shazia Jamshed

**Affiliations:** 1 Cyntax Health Projects, Contract Research Organization & Corporate Firm, Islamabad 45000, Pakistan; madeehamalik15@gmail.com (M.M.); ayishahashmi91@gmail.com (A.H.); 2 Hamdard Institute of Pharmaceutical Sciences, Hamdard University Islamabad, Islamabad 74600, Pakistan; humaira.rehman786@gmail.com; 3 Institute of Applied Sciences and Technology, Pak-Austria Fachhochschule, Haripur 22620, Pakistan; azharhussain10971@gmail.com; 4 Clinical Pharmacy and Pharmacy Practice, Royal College of Medicine Perak-Universiti Kuala Lumpur (UniKL), Ipoh 30450, Malaysia; khalid.sunaidar@unikl.edu.my; 5 Department of Oral Biology and Experimental Dental Research, Faculty of Dentistry, University of Szeged, 6720 Szeged, Hungary; balgeo@outlook.hu; 6 Doctoral School of Experimental and Preventive Medicine, University of Szeged, 6720 Szeged, Hungary; 7 Department of Pharmacy Practice, School of Pharmacy, IMU (International Medical University), Kuala Lumpur 57000, Malaysia; shaziajamshed@imu.edu.my; 8 Department of Pharmacy Practice, Jinnah University for Women, Karachi 74600, Pakistan

**Keywords:** anxiety, coping self-efficacy, cross-sectional survey, depression, mental health, urban setting

## Abstract

**Background/Objectives**: Mental health conditions represent a growing global health concern, disproportionately impacting populations in low- and middle-income countries like Pakistan. Limited epidemiological data, coupled with recent socioeconomic and environmental disruptions, has intensified the need for current insights into psychological burden and coping capacities in the Pakistani population. **Methods**: A descriptive, cross-sectional survey was conducted from January to May 2023 among 400 community-dwelling adults attending outpatient departments in Islamabad and Rawalpindi. A structured 75-item questionnaire incorporating validated tools (PHQ-9, GAD-7, WHO-5, CSES, and SRQ-20) was used to assess depression, anxiety, well-being, coping self-efficacy, and mental distress. Descriptive statistics, χ^2^ and Fisher’s exact tests, and Spearman’s rank correlation (r_s_) analyses were performed using IBM SPSS 22.0. **Results**: Most respondents were male (73.0%), aged 25–34 (60.0%), and urban-dwelling (80.0%). Clinically relevant depression and anxiety were observed in 57.0% and 19.5% of participants, respectively; 38.0% reported mental distress. Conversely, 76.5% demonstrated fair-to-good coping efficacy and 51.0% had high well-being scores. Younger age (≤34 years), higher income, urban residence, and male gender were associated with significantly better mental health outcomes. Strong positive correlation was found between PHQ-9 and GAD-7 scores (r_s_ = 0.672), and moderate negative correlations were found between GAD-7 and WHO-5 (r_s_ = −0.496), and PHQ-9 and WHO-5 (r_s_ = −0.310). **Conclusions**: Our findings highlight the significant psychological burden among urban Pakistani adults, alongside promising levels of resilience and coping self-efficacy. These results emphasize the urgent need for early, culturally adapted mental health screening and intervention programs in outpatient settings. Integrating such strategies into primary care, particularly for vulnerable subgroups like women, older adults, and those with lower income could facilitate timely diagnosis, improve outcomes, and reduce stigma surrounding mental health.

## 1. Introduction

Mental health conditions (including disorders such as generalized anxiety disorder, mood disorders, like major depressive disorder, bipolar disorder, obsessive-compulsive disorder, psychosomatic disorders, post-traumatic stress disorder, and schizophrenia, among others) constitute some of the most important burdens of disease worldwide, being a major factor in decreased quality of life (QoL) [1]. Based on the estimation of the World Health Organization (WHO) for 2019, around 1 billion people were living with various mental disorders globally [2]. Furthermore, based on Global Burden of Disease (GBD) estimates, the prevalence of mental disorders increased by 48.1% (from 654.8 million [1990] to 970.1 million [2019] cases), while disease burden (expressed as disability-adjusted life years (DALYs)) increased from 80.8 million DALYs to 125.3 million DALYs, becoming the seventh leading cause of disability by 2019 [1]. Furthermore, mental illnesses are often compounded with comorbidities, such as alcohol dependence or other substance use disorders, epilepsy, dementia, or other central nervous system issues, leading to complex neuropsychiatric issues that are difficult to manage [3]. Mental illnesses may affect people of all ages, genders, marital and family status, ethnic groups, religions, and educational and/or professional backgrounds; nonetheless, vulnerable patient groups are affected at disproportionate rates [4]. As these mental health disorders constitute a major risk for suicidal ideation and suicide attempts, their inadequate management constitutes a critical public health crisis globally [5]. Recent studies underscore how modern environmental behaviors, particularly among youth, are contributing to worsening mental health outcomes. A systematic review by Cangelosi et al. (2025) highlights a strong association between excessive Internet use and anhedonia, a core symptom of depression, marked by a reduced ability to experience pleasure [6]. Their summary of the available evidence has found that social anhedonia and feelings of loneliness are closely linked to Internet addiction and impaired social functioning in adolescents and young adults. These issues have been exacerbated by the novel coronavirus (COVID-19) pandemic, which intensified digital reliance due to prolonged isolation. Disruptions in sleep, reduced physical activity, and limited in-person interactions—consequences of excessive screen time—further diminish emotional resilience and well-being. The study emphasized the need to address these behavioral and environmental factors in youth-focused mental health strategies to prevent long-term psychological harm [6].

Approximately 80% of individuals with mental illnesses are residents of low and middle-income countries (LMICs), with 80% of patients with severe mental conditions in these regions having inadequate accessibility to the required mental health services [7]. Many LMICs have insufficient human capital for mental health services, and shortages are likely to persist; not only are mental health services scarce, but they are also inequitably distributed across counties, regions, and communities [8]. In addition, mental health disorders are associated with considerable stigma and societal taboo, limiting the utilization of available healthcare services [9]. The incidence and prevalence of mental disorders are increasing at an alarming rate in Pakistan. As mental health is still not considered a priority by health policy initiatives, strategies for screening and prevention of mental disorders have not been extensively implemented; moreover, as these conditions are associated with a social taboo, most of the cases go unreported or are undiagnosed and there is no vital registry of patients available. Although the exact epidemiological situation is still not known, it has been suggested that around 50 million people suffer from any one of the mental health issues in Pakistan [10]. In addition, enormous costs in terms of QoL, disability, and mental health conditions may result in severe direct economic losses [11,12]. Even still, less than half of Pakistan’s population who are affected by mental health issues have access to mental healthcare services; furthermore, only 0.4% of the government’s healthcare budget is allocated to mental health services, and of these funds, only 11.0% is dedicated to mental hospitals [1,4]. There is also a limited number of psychiatrists and allied mental health professionals to treat affected patients. In addition to financial constraints, lack of confidence in available modes of mental health treatment, previous personal experience, carelessness for mental disorders, religious fatalism, social stigma, individual ethnic groups, constraints by family, and dread of treatment have been identified as major barriers contributing towards the mental health disease burden in Pakistan [13,14]. As a consequence of all of the above, it has been estimated that between 130,000 and 270,000 persons commit suicide in Pakistan, with the majority of these individuals being young adults [15]. In addition to an already complex situation, the mental health status of the Pakistani population has been further compounded by recent events: on the one hand, numerous studies have underscored the detrimental impact of the COVID-19 pandemic on mental health, associated with lockdowns and the resulting isolation and frustration [16,17]. Furthermore, the pandemic has also resulted in a severe economic downturn—which was also influenced by a lack of political stability—leading to a severe economic crisis, inflation, loans, and a decline in living standards [18]; high levels of socioeconomic distress are also expected to cause additional mental health burden, as described by previous reports [19]. Finally, in 2022, Pakistan was subjected to an unprecedented environmental catastrophe due to flooding, affecting >30% of the land mass of the country, causing immeasurable economic damage, and displacing hundreds and thousands of people [14].

Standardized self-report instruments offer a pragmatic approach to identifying mental health concerns in community and clinical populations. The Patient Health Questionnaire (PHQ-9) is widely used for screening and grading depressive symptoms, while the Generalized Anxiety Disorder Scale (GAD-7) provides a brief, validated measure for anxiety severity. The WHO-5 Well-Being Index assesses positive mental well-being and is considered a reliable inverse indicator of emotional distress. The Coping Self-Efficacy Scale (CSES) measures individuals’ confidence in their ability to manage stress, while the Self-Reporting Questionnaire (SRQ-20), developed by the WHO, captures somatic and psychological symptoms of distress, which is especially relevant in LMIC settings. However, despite the availability of these tools, there is limited evidence of their application in outpatient populations in Pakistan. Most studies have focused on hospital inpatients or university students, leaving a critical gap in our understanding of mental health status, coping capacity, and associated socio-demographic determinants among general outpatient visitors—a group often overlooked but regularly interfacing with the health system. The aim of the present study was to address this theoretical and empirical gap by applying five validated instruments (PHQ-9, GAD-7, WHO-5, CSES, and SRQ-20) in a single framework to assess the psychological burden and coping strategies among urban Pakistani adults attending outpatient departments (OPDs). By exploring the interrelationships between these measures and identifying key demographic risk factors, this study provides a comprehensive, evidence-based foundation for integrating routine mental health screening in primary care. This study contributes to the scientific literature by (a) contextualizing the use of global screening tools in a South Asian outpatient population, (b) highlighting demographic risk patterns, and (c) informing culturally sensitive mental health strategies within the Pakistani healthcare system.

### 1.1. Primary Objectives

The main aim of this study was to assess the prevalence of depression, anxiety, mental distress, and overall psychological well-being among adults visiting outpatient departments (OPDs) in Islamabad and Rawalpindi.

### 1.2. Secondary Objectives

- To assess the role of various socio-demographic correlates (e.g., age, gender, income, residence, and education) in the prevalence of mental health outcomes, such as depression, anxiety, mental distress, and well-being.
- To determine correlations between standardized mental health instruments (PHQ-9, GAD-7, WHO-5, SRQ-20, and CSES, respectively).
- To identify demographic subgroups at elevated risk for poor mental health outcomes and reduced coping capacity.

## 2. Materials and Methods

### 2.1. Study Design, Setting, and Duration

A descriptive, cross-sectional, questionnaire-based study was carried out to determine the psychological burden and coping strategies of the Pakistani population in the so-called “twin-cities” of Islamabad and Rawalpindi, located in the Punjab province of Pakistan [20,21]. The study employed a convenience sampling approach to select the respondents willing to participate and available at the time of the data collection period [22]. Data collection was performed between 1 January 2023 and 31 May 2023.

### 2.2. Study Population, Inclusion and Exclusion Criteria

Data collection was carried out at OPDs in primary, secondary, and tertiary healthcare facilities (HCFs) situated in Islamabad (population in the metropolitan area ~2.36 million inhabitants, area: 906.5 km^2^ [23]) and Rawalpindi (population in the metropolitan area ~2.48 million inhabitants, area: 479.0 km^2^ [23]). These facilities included thirteen tehsil (i.e., local units of administrative division) and district headquarter HCFs in the division. The inclusion criteria for the study population included the following: (i) community-dwelling adults between the ages of 18 to 65 years, (ii) having a minimum of primary education, (iii) individuals who had the mental capacity to consent to medical treatment and/or participation in the study, (v) not pregnant, (vi) able to communicate in Urdu, (vii) visiting the OPDs for a routine medical check-up or treatment, and (viii) willing to take part in the study. The exclusion criteria included the following: (i) people who did not wish to take part in the study, (ii) those who were unable to understand the language used during data collection, and (iii) individuals who had a current or past medical history of mental illnesses [20,21].

The required sample size for the study was determined using the Raosoft Sample Size Calculator (http://www.raosoft.com/samplesize.html; accessed on: 1 July 2022), based on the formula described below (1):

(1) $$n=N\frac{x}{\left(N-1\right)E2+x}$$

where “x” is the expected response rate and “E” is the margin of error. The population was set to N = 20,000 (default setting of the software); the required confidence level was 95%; the acceptable margin of error was 5%; and the expected response rate was 50% [20]. Overall, a minimum sample size of n = 377 was set for the completion of this study.

### 2.3. Data Collection Tool, Validated Instruments Used

A paper-based, interviewer-administered, 75-item questionnaire was used for data collection, which included items corresponding to socio-demographic information and five validated instruments relevant to measuring concepts related to psychological burden and coping strategies. Socio-demographic information was collected pertaining to the participants’ sex, age, marital status, highest level of educational attainment, occupational status and profession, residence, and income bracket, respectively. Furthermore, the 9Item Patient Health Questionnaire (PHQ-9), the 7-Item Generalized Anxiety Disorder Scale (GAD-7), the WHO-5 Well-Being Index (WHO-5), the Coping Self-Efficacy Scale (CSES), and the WHO Self-Reporting Questionnaire (SRQ-20) were included in the questionnaire; permission had been obtained from the respective organizations to use the survey tools prior to undertaking this study. All instruments utilized for this study were already validated and available in Urdu language versions, which were included in the final version of the questionnaire. To ensure the reliability and validity of the data collection tools, pilot testing was conducted on 10% of the expected sample (*n* = 38), which was not included in the analyzed sample [21].

The PHQ-9 was used to assess depressive symptoms among respondents related to their experiences over the past 2 weeks, corresponding to nine items related to depressive symptomatology [22]. The scoring of PHQ-9 takes into account the sum of all the responses from the participants, with each item scored on a scale of 0–3 (0 = not at all; 1 = several days; 2 = more than a week; and 3 = nearly every day), with a total score ranging from 0 to 27; higher scores reflect worse depressive symptoms. Scores may be classified as follows: 0–4 is classified as no depression, 1–4 as minimal depression, 5–9 as mild depression, 10–14 as moderate depression, 15–19 as moderately severe depression, and ≥20 as severe depression, respectively. In the analysis, the results were dichotomized as “not depressive” (scores 0–4) and “depressive” (5–27) [23]. Cronbach’s alpha (α) of the instrument based on the pilot testing results was 0.71. The GAD-7 was used to assess generalized anxiety symptoms in respondents over the past 2 weeks, corresponding to seven items related to generalized anxiety symptoms [24]. The scoring of the GAD-7 involves each item being a four-point Likert scale (0–3) with total scores ranging from 0 to 21; higher scores reflect greater anxiety severity. The scores may be classified as follows: 0 as no anxiety, 1–4 as minimal anxiety, 5–9 as mild anxiety, 10–14 as moderate anxiety, and 15–21 as severe anxiety, respectively. In the analysis, the results were dichotomized as “anxiety below the clinical range” (scores 0–9) and “anxiety in the clinical range” (10–21) [23]. Cronbach’s α of the instrument is 0.69. The WHO-5 was used to assess positive psychological well-being in respondents over the past 2 weeks [25]. The scoring of the WHO-5 includes a sum of all the responses on a 6-point Likert scale, ranging from 0 (at no time) to 5 (all of the time); raw scores were transformed to a score from 0 to 100, with higher scores indicating better well-being and lower rates of mental health issues. Scores were classified as follows: 0–25 as very poor well-being, 26–50 as poor well-being, 51–75 as satisfactory well-being, and 76–100 as good well-being, respectively. According to the literature, a score of ≤50 indicates poor well-being and suggests further investigation into possible symptoms of depression, while a score ≤ 28 is indicative of depression [26]. In the analysis, the results were dichotomized as “low or satisfactory well-being” (scores 0–75) and “good well-being” (76–100) [23]. Cronbach’s α of the instrument based on the pilot testing results was 0.74. The CSES was used to assess the perceived ability and confidence of the participants to carry out certain actions, measured on a 26-item scale [27]. The scoring includes the sum of the responses on a scale of 0 (“cannot do at all”), 5 (“moderately certain can do”), and 10 (“certain can do”); the maximum score achievable is 260, where higher points indicate more confidence in the individual’s ability to carry out certain actions. In the analysis, the results were dichotomized as “mild coping self-efficacy” (scores 0–129) and “fair or good coping self-efficacy” (130–260) [23]. Cronbach’s alpha α of the instrument based on the pilot testing results was 0.78. Finally, the SRQ-20 instrument comprises 20 items and was used to assess psychological and somatic symptoms in the past 30 days [28]. The scoring includes the sum of the responses on a scale scored 0 or 1, with a score of 1 indicating that the symptom was present during the last month, while a score of 0 indicates that the symptoms were not present. Scores ≤ 9 were classified as “no mental distress”, while scores > 9 were classified as “mental distress”, respectively [23]. Cronbach’s alpha α of the instrument based on the pilot testing results was 0.72.

### 2.4. Data Collection Process

All interviews were conducted during visiting hours at the OPDs, in the waiting rooms of the HCPs. After comprehensively informing the participants of the study objectives and obtaining consent, respondents were interviewed separately from other patients at the OPDs, in a private room. To avoid bias, questionnaires were interviewer-administered [29]. If the participant was required by the medical personnel in the OPDs, then the interviewer suspended the interview and continued after the patient completed the required medical visit.

### 2.5. Statistical Analysis

Following the interviews, questionnaire data were entered into spreadsheets (Microsoft Excel; Microsoft Corp. Redmond, WA, USA), and then transferred to Statistical Package for Social Sciences v.22.0 (SPSS; IBM Corp., Endicott, NY, USA) for analysis. During descriptive statistics, all continuous variables were expressed as medians (with range), while categorical variables were described as frequencies and percentages (*n*, %). Normality testing was performed using the Q-Q diagram and Kolmogorov–Smirnov tests. Associations between categorical variables were assessed using χ^2^-tests and Fisher’s exact tests, with Cramér’s V effect size measure [φ], in case of significant results; on the other hand, correlation among numerical variables was assessed using Spearman’s rank correlation coefficients (r_s_). The strength of the relationship between variables was described as follows: |r_s_| < 0.3 was denoted as a weak correlation, 0.3 < |r_s_| < 0.5 as a moderate correlation, and 0.5 < |r_s_| < 0.85 as a strong correlation [30]. During analyses, *p*-values < 0.05 were considered statistically significant.

### 2.6. Ethical Considerations, Consent

This study was conducted in accordance with the Declaration of Helsinki (1975, last revised in 2013), and national and institutional ethical standards. Ethical approval for this study was obtained from the Ethical Committee of Hamdard University (IRB# 345). Ethical approval was also obtained from medical superintendents of the healthcare facilities located in Islamabad and Rawalpindi, where data collection was conducted during the study period.

Before participating in this study, each individual was informed about the nature and objectives of the study, privacy, anonymity, and confidentiality of their data, and both verbal and written informed consent was obtained from the patients who agreed to participate before data collection. Participants were made aware that their participation in the research is voluntary, and they may withdraw from the study at any time without any consequences. The participants did not receive any incentives (monetary or otherwise) to take part in the study.

### 2.7. Reporting Standards

This manuscript adheres to the STROBE (Strengthening the Reporting of Observational Studies in Epidemiology) guidelines for cross-sectional studies to ensure methodological rigor, transparency, and reproducibility [31]; the STROBE checklist is provided in Supplementary Material S1. The use of this structured reporting framework supports the clarity and consistency of our observational study [31].

## 3. Results

### 3.1. Response Rate, Socio-Demographic Characteristics

To ensure the required number of participants, *n* = 480 individuals were approached to take part in our study, out of which, *n* = 400 (response rate: 83.3%) agreed to respond (resulting in a margin of error: 4.85% < 5%). The majority of participants were males (*n* = 292, 73.0%) between the ages 25 to 34 years (*n* = 240, 60.0%; median age: 28 years, range: 18–54), residing in an urban area (*n* = 320, 80.0%), employed (*n* = 356, 89.0%), earning at least PKR 19,000 (Pakistani Rupees) per month, USD ~70 (US dollars), and had at least a bachelor (BSc/BA) degree (*n* = 396, 99.0%). A detailed summary of the socio-demographic characteristics of the participants is shown in Table 1. The professions of the participants and their distribution were as follows: accountant 9.0% (*n* = 36), business and business administration 4.5% (*n* = 18), government employee 10.0% (*n* = 40), graphic designer 1.0% (*n* = 4), lawyer 1.0% (*n* = 4), microbiologist 2.5% (*n* = 10), nutritionist 2.0% (*n* = 8), pharmacist 30.0% (*n* = 120), physician 2.0% (*n* = 8), police officer 6.0% (*n* = 24), press 2.0% (*n* = 8), sales and marketing 4.0% (*n* = 16), student 8.0% (*n* = 32), and teacher/educator 18.0% (*n* = 72), respectively. In subsequent analyses (i.e., χ^2^-tests and Fisher’s exact tests), the following categories were compared: gender (male vs. female), age (up to 34 years vs. above 34 years), marital status (single vs. individuals who are or have been married), place of residence (urban vs. rural), highest level of education (up to BSc/BA vs. above BSc/BA), and income level/month (up to PKR 19,000 vs. above PKR 19,000). Due to the low number of participants in some subcategories, employment was excluded from subsequent analysis.

### 3.2. Psychological Burden and Coping in Participants

Based on the scores obtained by the respondents from the PHQ-9 instrument and relevant cut-off values, our respondents were classified as follows: 14.0% (*n* = 56) no depression, 29.0% (*n* = 116) minimal depression, 36.0% (*n* = 144) mild depression, 10.5% (*n* = 42) moderate depression, 6.5% (*n* = 26) moderately severe depression, and 4.0% (*n* = 16) severe depression, respectively (median PHQ-9 score [range]: 9 [1–25]). In further analysis, the results were dichotomized as “not depressive” (43.0%, *n* = 172) and “depressive” (57.0%, *n* = 228). According to the GAD-7 anxiety severity scores, the respondents were classified as follows: 18.5% (*n* = 74) no anxiety, 30.0% (*n* = 120) minimal anxiety, 32.0% (*n* = 128) mild anxiety, 11.0% (*n* = 44) moderate anxiety, and 8.5% (*n* = 34) severe anxiety, respectively (median GAD-7 score [range]: 6 [0–19]). In further analysis, the results were dichotomized as “anxiety below the clinical range” (80.5%; *n* = 322) and “anxiety in the clinical range” (19.5%; *n* = 78). Based on the scores from the WHO-5 instrument and cut-off values, 9.0% (*n* = 36) of participants were classified as having very low well-being, 10.5% (*n* = 42) as low well-being, 29.5% (*n* = 118) as satisfactory well-being, and 51.0% (*n* = 204) as good well-being, respectively (median WHO-5 score [range]: 80 [18–100]). In the analysis, the results were dichotomized as “low or satisfactory well-being” (49.0%; n = 196) and “good well-being” (51.0%; *n* = 204). According to the CSES results, 23.5% (n = 94) had mild coping self-efficacy, while 76.5% (*n* = 306) showed fair or good coping self-efficacy, respectively (median CSES score [range]: 211 [76–257]). Finally, based on the SRQ-20 results, 62.0% (*n* = 248) of the respondents presented with no mental distress, while 38.0% (*n* = 152) had scores indicating mental distress, respectively (median SRQ-20 score [range]: 5 [0–18]).

The results of univariate statistical analyses, comparing the socio-demographic characteristics of the participants and psychological burden and coping are summarized in Table 2. Analysis of socio-demographic characteristics revealed several associations with psychological burden and coping measures among the participants. Gender was significantly associated with anxiety and well-being, with females more likely than males to report anxiety in the clinical range (*p* = 0.001, φ = 0.304) and lower well-being scores (*p* = 0.002, φ = 0.233). Age was also significantly associated with psychological burden: participants aged 34 years or younger were more likely to report depressive symptoms (*p* = 0.003, φ = 0.312), anxiety in the clinical range (*p* = 0.006, φ = 0.246), and lower coping self-efficacy (*p* = 0.002, φ = 0.300). Marital status showed significant associations with depression and anxiety, where single individuals were more likely to report both depressive symptoms (*p* = 0.005, φ = 0.218) and anxiety in the clinical range (*p* = 0.006, φ = 0.258). Place of residence (urban vs. rural) did not show any significant association with any of the psychological burden or coping indicators. Regarding educational attainment, participants with education beyond a bachelor’s degree (BSc/BA) demonstrated significantly better coping self-efficacy (*p* = 0.001, φ = 0.315), although education level was not significantly associated with depression, anxiety, or well-being. Monthly income was a significant factor in depression and anxiety outcomes. Respondents with a monthly income of PKR 19,000 or below were significantly more likely to report depressive symptoms (*p* = 0.016, φ = 0.194) and anxiety in the clinical range (*p* = 0.002, φ = 0.401). However, no significant association was found between income and self-efficacy, well-being, or mental distress.

### 3.3. Correlation Analysis

In the context of correlation between the scales used to assess psychological burden and coping, a strong and significant positive correlation was observed between the results of the PHQ-9 and GAD-7 scales (r_s_: 0.672; *p* = 0.004); moderately strong and negative correlations were observed between GAD-7 and WHO-5 (r_s_: −0.496; *p* = 0.01) and PHQ-9 and WHO-5 (r_s_: −0.310; *p* = 0.014); weak and positive correlations were observed between WHO-5 and CSES (r_s_: 0.247; *p* = 0.043), GAD-7 and SRQ-20 (r_s_: 0.222; *p* = 0.040), and PHQ-9 and SRQ-20 (r_s_: 0.203; *p* = 0.048); and weak and positive correlations were observed between GAD-7 and CSES (r_s_: −0.276; *p* = 0.023) and WHO-5 and SRQ-20 (r_s_: −0.273; *p* = 0.035), respectively. A complete summary of the correlation results from our respondents’ data is shown in Table 3.

## 4. Discussion

### 4.1. Discussion of Our Findings

Mental health conditions are highly prevalent worldwide, yet often remain undiagnosed or undertreated, significantly impairing individuals’ quality of life (QoL) [32]. The WHO World Mental Health Survey Initiative indicates high lifetime prevalence rates for anxiety, mood, impulse control, and substance use disorders, with earlier onset seen in younger populations [33]. In Pakistan, where mental health infrastructure is underdeveloped, these disorders contribute to a large share of avoidable morbidity and mortality, particularly among the youth [1,3,12,15]. Although psychotropic medications are accessible, the lack of integrated mental health subspecialties, care continuity, and trained personnel inhibit comprehensive treatment [11]. This study contributes to the limited evidence on psychological burden and resilience among urban Pakistani adults. The findings demonstrate that 57% of participants had at least mild depressive symptoms and 19.5% experienced anxiety at clinical levels. These prevalence rates align with or exceed regional estimates and underscore the urgent need for community-level screening and early intervention. Notably, our results also reflect promising levels of coping self-efficacy (76.5%) and psychological well-being (51%), suggesting that resilience mechanisms may be prevalent even in challenging socioeconomic environments.

Demographic analyses showed that female gender, older age, and lower income were associated with worse mental health outcomes. These findings align with regional and global literature highlighting the vulnerability of women to depression and anxiety, due to sociocultural expectations, financial dependency, and limited autonomy [34,35]. This study also supports existing research that urban residence and higher educational attainment correlate with better coping efficacy and emotional well-being [36]. Correlations among standardized instruments further validate the robustness of these findings. The positive correlation between the PHQ-9 and GAD-7 scores and negative associations with WHO-5 is consistent with prior studies in both high- and low-income settings [22,37,38]. These patterns reinforce the utility of combining multiple validated tools for nuanced mental health assessment.

Our study also adds to evidence that self-perceived failure and lack of social support exacerbate anxiety and distress, which is consistent with Japanese and UK-based studies [39,40]. Conversely, high coping scores in our participants echo findings from studies in Guyana and Denmark, emphasizing the potential for community-based mental health promotion strategies [41,42]. The findings resonate with international work on the role of self-efficacy and emotional resilience in sustaining well-being [36]. Importantly, these insights must be interpreted within contextual limitations. Pakistan’s mental health stigma, spiritual interpretations of psychological distress, and collective familial norms heavily influence care-seeking behavior [43]. Mental health disorders are not only viewed through a biomedical lens but are often perceived as moral or spiritual failures, limiting engagement with formal healthcare services. These cultural barriers necessitate the inclusion of religious and community leaders in advocacy campaigns and require clinicians to engage with culturally congruent treatment narratives.

Overall, our findings underscore the critical gaps in mental health support in Pakistan and call for an expansion of culturally adapted, scalable interventions within outpatient and primary care settings. Screening tools such as the PHQ-9, GAD-7, and WHO-5 are simple yet powerful instruments that may be deployed with minimal training, allowing for earlier identification and timely referral. The results of this study have clear implications for outpatient and community-based healthcare delivery. The high burden of depressive and anxiety symptoms observed reinforces the necessity of implementing systematic mental health screening in primary care settings. Validated instruments such as the PHQ-9 and GAD-7 should be incorporated into routine outpatient assessments, particularly targeting high-risk groups including women, older adults, and individuals from lower-income brackets. Furthermore, the substantial proportion of participants exhibiting high coping self-efficacy and well-being scores suggests an opportunity to enhance protective factors through targeted interventions. Culturally sensitive psycho-educational programs, brief counseling, and community-based resilience-building initiatives may be integrated into the existing health infrastructure. Health professionals should be trained in stigma reduction, culturally competent communication, and early detection of common mental disorders. The development of referral networks linking primary care with mental health specialists is critical for improving care pathways and continuity. Finally, leveraging digital tools such as SMS-based screening or mobile apps may further improve access and engagement, particularly among younger urban populations. Such strategies should be complemented by efforts to normalize mental health dialogue through mass media and religious institutions, fostering a supportive environment for help-seeking behaviors.

### 4.2. Limitations

This study has several limitations that should be considered when interpreting the findings. Firstly, its cross-sectional design prevents any conclusions about causal relationships between mental health outcomes and associated factors. Secondly, the use of convenience sampling from outpatient departments in two urban centers (Islamabad and Rawalpindi) may introduce selection bias, limiting the representativeness of the findings. The participant profile of predominantly young, educated, and urban individuals does not reflect the broader national demographic and may underestimate the population-wide mental health burden. Additionally, self-reporting and interviewer bias may have influenced the results, though validated Urdu instruments and pilot testing helped mitigate this risk. Thirdly, while this study employed validated tools translated into Urdu, data collection relied on self-reported measures, which may be subject to response bias or underreporting due to the stigma surrounding mental health. Fourthly, the participant population was predominantly composed of young, educated, urban-dwelling males, which does not reflect the broader diversity of the Pakistani population in terms of gender, rural residence, socioeconomic status, or literacy levels. Therefore, the generalizability of the results to rural areas, lower-income communities, and other healthcare settings is limited. Future research using probability-based sampling across diverse geographic regions and health facilities, as well as longitudinal designs, would be essential for validating these findings and tracking mental health trends over time.

## 5. Conclusions

The present cross-sectional study identified a high burden of depressive symptoms (57.0%) and clinically significant anxiety (19.5%) among urban Pakistani adults attending outpatient departments, with substantial proportions also reporting mental distress. Despite this, high levels of coping self-efficacy (76.5%) and well-being (51%) suggest underlying resilience. Significant correlations observed between the PHQ-9, GAD-7, and WHO-5 scores reinforce the validity of using these tools in routine screening. The findings confirmed that female gender, older age, and lower income were significantly associated with poorer mental health outcomes, while urban residence and higher education were linked to better coping and well-being. These results underscore the need for targeted screening, culturally adapted interventions, and integration of validated instruments (PHQ-9, GAD-7, WHO-5, SRQ-20, and CSES) into primary care. Although limited by its cross-sectional design and an urban sample, this study provides critical baseline data. Future multi-site, longitudinal studies are warranted to enhance generalizability. In view of Pakistan’s growing mental health burden, scalable, evidence-based, and culturally sensitive mental health strategies are urgently required within outpatient and community health settings.

## Figures and Tables

**Table 1:** Summary of the demographic characteristics of the study participants.

Variable	Category	*n*	%
Gender	Male	292	73.00%
	Female	108	27.00%
Age group	18–24	64	16.00%
	25–34	240	60.00%
	35–44	92	23.00%
	45–54	4	1.00%
Marital status	Single	204	51.00%
	Married	192	48.00%
	Divorced/Widowed	4	1.00%
Residence	Urban	320	80.00%
	Rural	80	20.00%
Education	Primary/Secondary	4	1.00%
	Bachelors (BSc/BA)	152	38.00%
	Masters (MSc/MA)	232	58.00%
	Doctorate (PhD)	12	3.00%
Occupational status	Unemployed	44	11.00%
	Employed	356	89.00%
Monthly income (PKR)	None	44	11.00%
	≤19,000	16	4.00%
	19,001–50,000	148	37.00%
	50,001–100,000	120	30.00%
	≥100,001	72	18.00%

PKR: Pakistani Rupee; US: United States.

**Table 2:** Relationship between the socio-demographic characteristics of the participants and psychological burden and coping.

Variable	PHQ-9 (Depressive Symptoms)	GAD-7 (Anxiety)	WHO-5 (Well-Being)	CSES (Self-Efficacy)	SRQ-20 (Mental Distress)
Gender	Not significant (*p* = 0.096, φ < 0.120)	Males < Females **(*p* = 0.001, φ = 0.304)**	Males < Females **(*p* = 0.002, φ = 0.233)**	Not significant (*p* = 0.199, φ < 0.120)	Not significant (*p* = 0.168, φ < 0.120)
Age	≤34 yrs < depressive symptoms **(*p* = 0.003, φ = 0.312)**	≤34 yrs < anxiety **(*p* = 0.006, φ = 0.246)**	Not significant (*p* = 0.068, φ < 0.120)	≤34 yrs < low efficacy **(*p* = 0.002, φ = 0.300)**	Not significant (*p* = 0.169, φ < 0.120)
Marital status	Single > depressive symptoms **(*p* = 0.005, φ = 0.218)**	Single > anxiety **(*p* = 0.006, φ = 0.258)**	Not significant (*p* = 0.208, φ < 0.120)	Not significant (*p* = 0.735, φ < 0.120)	Not significant (*p* = 0.346, φ < 0.120)
Residence	Not significant (*p* = 0.697, φ < 0.120)	Not significant (*p* = 0.067, φ < 0.120)	Not significant (*p* = 0.146, φ < 0.120)	Not significant (*p* = 0.958, φ < 0.120)	Not significant (*p* = 0.383, φ < 0.120)
Education	Not significant (*p* = 0.227, φ < 0.120)	Not significant (*p* = 0.094, φ < 0.120)	Not significant (*p* = 0.100, φ < 0.120)	BSc+ > self-efficacy **(*p* = 0.001, φ = 0.315)**	Not significant (*p* = 0.340, φ < 0.120)
Income	≤ PKR 19,000 < depressive symptoms **(*p* = 0.016, φ = 0.194)**	≤ PKR 19,000 < anxiety **(*p* = 0.002, φ = 0.401)**	Not significant (*p* = 0.195, φ < 0.120)	Not significant (*p* = 0.707, φ < 0.120)	Not significant (*p* = 0.184, φ < 0.120)

The abbreviations of the psychological burden and coping strategies are found in the text. Analyses were carried out using χ^2^-tests and Fisher’s exact tests, with Cramér’s V effect size measures [φ]. Relational signs (<, >) describe the direction of the relationship. *p*-values below 0.05 are shown in **boldface**.

**Table 3:** Correlation coefficients (r_s_) between measures of psychological burden and coping of the participants.

	PHQ-9	GAD-7	WHO-5	CSES	SRQ-20
**PHQ-9**		**0.672** **(*p* = 0.004)**	**−0.310** **(*p* = 0.014)**	−0.187 (*p* = 0.155)	**0.203** **(*p* = 0.048)**
**GAD-7**	**0.672** **(*p* = 0.004)**		**−0.496** **(*p* = 0.01)**	**−0.276** **(*p* = 0.023)**	**0.222** **(*p* = 0.040)**
**WHO-5**	**−0.310** **(*p* = 0.014)**	**−0.496** **(*p* = 0.01)**		**0.247** **(*p* = 0.043)**	**−0.273** **(*p* = 0.035)**
**CSES**	−0.187 (*p* = 0.155)	**−0.276** **(*p* = 0.023)**	**0.247** **(*p* = 0.043)**		−0.135 (*p* = 0.214)
**SRQ-20**	**0.203** **(*p* = 0.048)**	**0.222** **(*p* = 0.040)**	**−0.273** **(*p* = 0.035)**	−0.135 (*p* = 0.214)	

The abbreviations of the psychological burden and coping strategies are found in the text. *p*-values below 0.05 are shown in **boldface**.

## Data Availability

All data generated during the study are presented in this paper.

## Supplementary Materials

The following supporting information can be downloaded at https://www.mdpi.com/article/10.3390/epidemiologia6030030/s1: Supplementary Material S1: STROBE checklist.
